# A review of the impact of social disruptions on food security and food choice

**DOI:** 10.1002/fsn3.3752

**Published:** 2023-10-12

**Authors:** Claire D. Munialo, Duane D. Mellor

**Affiliations:** ^1^ Food, Land and Agribusiness Management Harper Adams University Newport UK; ^2^ Aston Medical School Aston University Birmingham UK

**Keywords:** conflicts, consumer behavior, food choice, food security, global pandemic, recession

## Abstract

At times of severe social disruption, be that war, conflict, pandemic, or economic downturn, both the availability and consumption of healthy dietary patterns can be adversely affected with any effects often outlasting the initial social disruption. For instance, when the COVID‐19 pandemic hit and lockdown restrictions followed, households were reported to consume unhealthy diets. In some cases, this seemed to be a response to the situation and a coping mechanism. In contrast, in other cases, it was a consequence of limited food availability or access, with some communities finding that fresh foods became difficult to source due to the disruption in global supply chains. The example presented by the impact of conflict in Ukraine, which has also disrupted global food supply chains, at a macrolevel, food systems and at a microlevel, individual and community shows that food choices may respond to different global events in similar ways. Therefore, in this review, a range of events/disruptions are considered, beyond pandemics and wars, including climate disasters (e.g., fire, famine, and floods) that have been shown to impact food supply and consequently, food security. The importance of this can be seen as inadequate and nutritionally poor diets have a concomitant effect on health, which extends beyond the initial period of societal stress and disruption of food supply chains. Hence, the impact of such disruptions on consumer behavior which includes food choice needs to be corroborated. Therefore, this review aims to discuss the impact of such disruptions on consumer behavior and food choices. Additionally, this review provides some practical strategies that can be used to ensure the availability of healthy diets.

## Graphical abstract

Severe social disruption affects the availability and consumption of healthy dietary patterns. Globally, the causes of food insecurity are diverse. Disruption in global supply chains limits food availability or access. The global community needs to see access to food as a key human right.

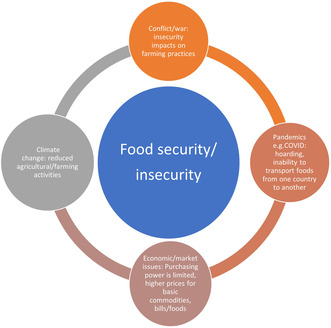

## 1 INTRODUCTION

Dietary intakes are highly associated with health outcomes, with poor dietary patterns being associated with increased risk of chronic diseases including cardiovascular disease, type 2 diabetes, a number of cancers (Roberts & Barnard, 2005), and early mortality (Wang, et al., 2019). The major factors shaping dietary intake and patterns include the availability of food, socioeconomic status, culture, beliefs, and preferences among others (Laska et al., 2010). When considering factors which influence food intake, there are several concepts around food choice and security which this review will consider, these are defined in Table 1.

**TABLE 1:** Summary of definitions of terms relating to food choice, availability, and security.

Term	Definition	Reference
Food security	This is the availability (at all times) of “adequate world food supplies of basic foodstuffs to sustain a steady expansion of food consumption and to offset fluctuations in production and prices”	Pinstrup‐Andersen (2009)
Food preferences	These are the evaluative attitudes that individuals express toward foods	Birch (1999)
Food choice	This is the access to adequate resources for acquiring appropriate foods for a nutritious diet by individuals	Larson and Story (2009)
Famine	This is the widespread scarcity of food which can be the result of several factors such as crop failure, war, natural disasters, an economic catastrophe, widespread poverty, or government policies	Scrimshaw (1987)
Food availability	This is the availability of food in sufficient quantities and of appropriate quality, which is supplied through domestic production or imports (including food aid)	Burchi and De Muro (2016)

Several factors contribute to or influence changes in dietary intake and consumer behavior. Consumer behavior plays an important role in buying behavior and food choice and these can be impacted by several factors that include the “so‐called three lethal C's” which are the COVID‐19 global pandemic, Conflict in various zones, and changes in the Climate (More et al., 2020). These factors have a concomitant impact on food demand and supply as the number of people in food crises continues to rise with several people across many countries in the world being affected. A summary of the number of people in food crisis or worse classified by primary driver/disruption between 2018 and 2020 is shown in Table 2.

**TABLE 2:** A summary of the number of people in food crisis or worse as classified by primary driver/disruption between 2018 and 2020. Adapted from (Crises, 2021).

Primary driver	2018	2019	2020
Economic Shocks	10.2 M in 6 countries	23.9 M in 9 countries	40.5 M in 17 countries
Climate (Weather events)	28.7 M in 26 countries	33.7 M in 25 countries	15.7 M in 15 countries
Conflict/insecurity	73.9 M in 21 countries	77.1 M in 22 countries	99.1 M in 23 countries

The recent World Food Program report reported around 258 million people in 58 countries and territories faced acute food insecurity at crisis or worse levels in 2022, which was up from the reported 193 million people in 53 countries and territories in 2021 (World Food Programme, 2023a). In 2023, a total of 43.3 million people across 51 countries have been reported to be in emergency or greater levels of acute food insecurity (World Food Programme, 2023b) some of which is the consequence of the disruptions listed in Table 2. The impact of such disruptions includes the availability of food, and this poses a food security challenge. Thus, to understand the impact of various factors on food security, there is a need for the various disruptions to be explored and their impact on consumer behavior and food choices to be understood. According to the World Food Summit (1996), “food security exists when all people, at all times, have physical and economic access to sufficient, safe and nutritious food that meets their dietary needs and food preferences for an active and healthy life” (Summit, 1996).

There is a lot to learn from how countries, communities, and individuals respond to various international societal stressors and crises. Several commentators and researchers have explored the impact of a range of global events including economic crises (Basen, 2014), a global pandemic (Galanakis, 2020), or conflict and war (Osendarp et al., 2022; Sassi, 2021) on food availability and insecurity. However, there is a paucity of knowledge on the impact of all these factors on food options, choice, and behaviors and a concomitant impact on nutrition and health. Given the past few years where global events appear to have moved from one disaster or crisis, which rapidly have been replaced by another and so on, there is a need to look at all these factors in combination rather than in isolation and to research practical ways that the public can adopt to ensure healthy living. Therefore, this review discusses the impact of such disruptions on consumer behavior and food choices. Given the fact that such disruptions are interconnected and their impact on food supply is not mutually exclusive, these factors will be reviewed in combination rather than in isolation. Additionally, this review provides some practical strategies that can be used to ensure the availability of healthy diets.

## 2 FACTORS THAT CAN DISRUPT THE FOOD SYSTEMS AND IMPACT FOOD SECURITY

Several factors contribute to or influence changes in dietary intake and consumer behavior. These factors can broadly be classified as outlined in Table 3 and ultimately manifest as how consumer's emotions, attitudes, and preferences affect the food choices of individuals, families, and communities.

**TABLE 3:** An outline of key factors that influence/determine changes in dietary intake and consumer behavior.

Factor	Examples	Remark	Reference
Sociological	Buying habits, level of income, and family size and structure	These factors develop and influence food choice at a macrolevel and are also linked to cultural and social norms	National Research Council (2013)
Psychological	Perceptions of hunger, satiation, and appetite	These factors can generally be more influenced by individuals	Stavkova et al. (2008)
Marketplace (economic)	Income, the cost of food, and the education level of the head of the household	These factors can stress household budgets and have been used, e.g., as indicators for hunger and the likelihood of food insufficiency	Rose (1999)

The sociological, psychological, and economic factors described in Table 3 can be affected by several aspects such as conflict/war, climate changes as well as unprecedented happenings such as the outbreak of pandemics all of which have the ability to impact consumer behavior and food choices and will be discussed in subsequent sections.

### 2.1 COVID‐19 pandemic and its impact on food security and food choices

The emergence of the coronavirus pandemic exacerbated the existing discrepancies in the food sector, and this led to a significant destabilization of the global food security system. In general, the food systems comprise multifaceted closely correlated stages that are meant both to ensure the traceability of the final product from farm to fork traceability as well as: (i) the security of the final products during processing, distribution, and consumption, (ii) the activities that are aimed at waste management, and (iii) the maintenance of a connection between all involved parts (Galanakis, 2020).

**FIGURE 1:**
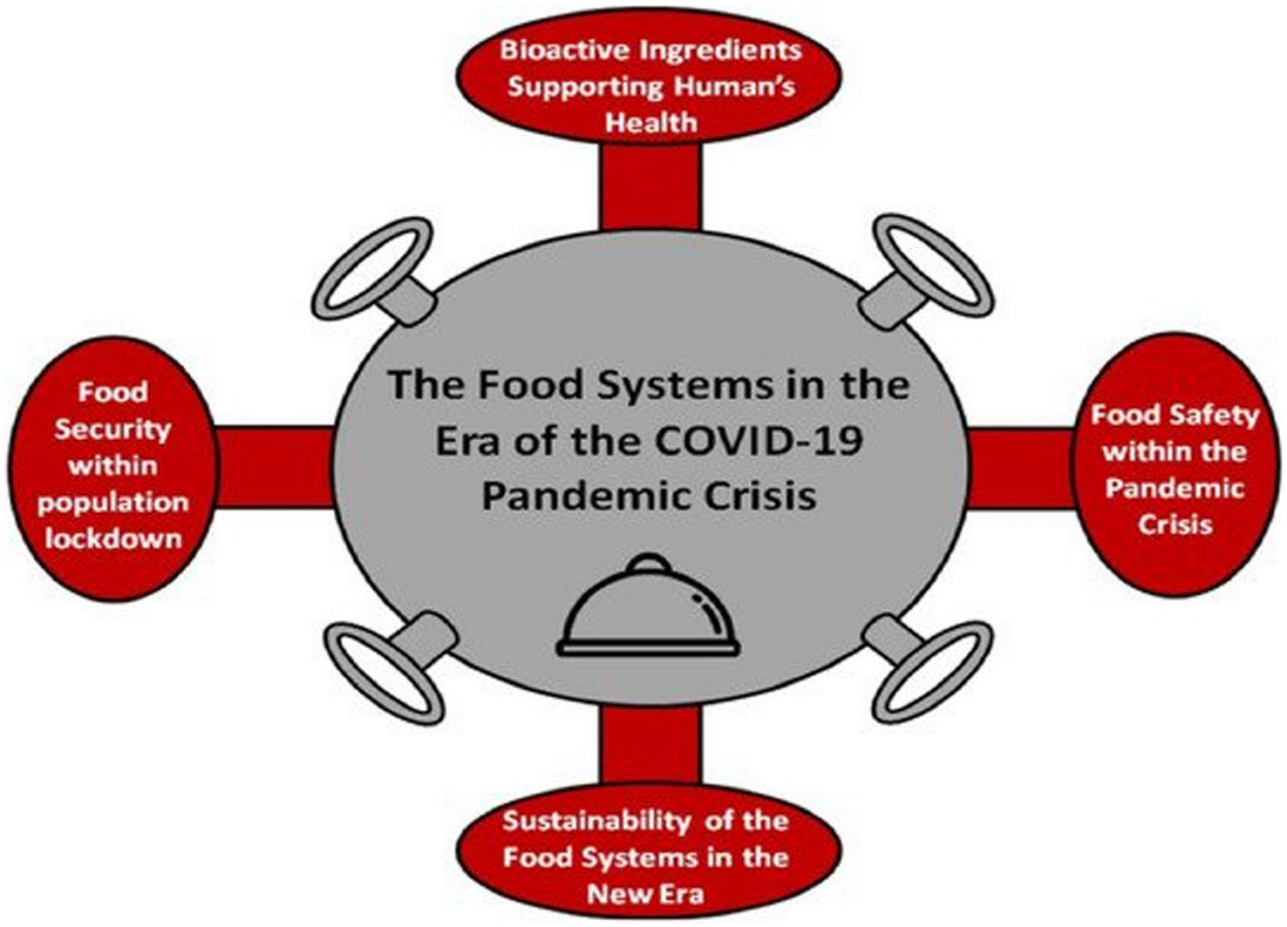
The food systems in the era of the coronavirus disease (COVID‐19) pandemic crisis (Galanakis, 2020). The use of this figure is allowed under Creative Commons license: https://creativecommons.org/licenses/by/4.0/.

The four significant issues that the food industry has had to address in response to the pandemic include: (i) the desire of the consumers in developed economies to protect themselves and their immune system and this contributed to some individuals adopting healthier diets. Consequently, the availability of bioactive ingredients of food and functional foods such as bioactive peptides, antioxidants, antimicrobials, immunomodulatory, anti‐inflammatory, and antithrombotic compounds (Galanakis et al., 2020) which can be found in fruits and vegetables has become critical, as the demand for these products increased. (ii) food safety was viewed to be a significant issue to avoid the spreading of the virus between producers, retailers, and consumers. This has resulted in some small‐ to medium‐scale producers not being able to return to full‐scale production; with several employees being on sick leave or laid off work and this has had an impact on the supply of some food commodities. (iii) food security issues that emerged due to the lockdown of a billion people inside their houses, which was seen initially in the form of several individuals ‘panic buying’ of nonperishable foods (Lehberger et al., 2021), linked to perceptions of social norms, anxiety about availability, and concerns linked to missing out (Prentice et al., 2022), and (iv) the sustainability of the food systems in the era of pandemics and this is an issue that the food sector should address to restrict relevant crises in the future, including the potential need to introduce restrictions on consumers including rationing either by retailers and/or by government agents (Hobbs, 2020). These issues are summarized in Figure 1.

If the impact of the global pandemic was not enough on the availability of food commodities, the aftermath of the lockdown, which includes a global increase in the cost of production and food prices, and a rise in global demand have combined and this has continued to add a squeeze on already stressed household incomes. This has affected the availability of healthy foods for consumers. For example, a recent analysis in the UK by the Office of National Statistics (Casey et al., 2022) revealed the increase in the cost of some items to be up to 50% more. The rise in the cost of living, the socioeconomic differences between consumers, and the unequal distribution of resources (in different regions around the world) are generating discrepancies in living standards with food poverty and food insecurity increasing.

#### 2.1.1 How COVID‐19 influenced food choices and behavior

The food supply and demand channels (which indicate a decrease in food stock and a rise in food prices) were both directly affected by the pandemic, whereas the purchasing power and the ability to produce and distribute food through the normal logistic supply chains were indirectly affected by the pandemic, with the vulnerable population groups (which are generally women, older adults, and children) and the poor being the most affected both in the developing (Panthi et al., 2020) and developed countries (Miguel & Mobarak, 2022; Schellekens & Sourrouille, 2020). As such, the consumption of nutritious food can tend to be reduced, and this ‘deficit’ can then be replaced with the consumption of nutritionally deficient foods. For instance, an increase in food prices due to the combined effects of post‐COVID increase in energy demand has the potential of resulting in a reduction in intake of fruit and vegetables, which can impact dietary patterns making them less healthy in general. Fruits and vegetables are considered to be key dietary components required to adhere to many nation's dietary guidance as they do make an important contribution to the intakes of dietary fiber, vitamins, minerals, as well as phytochemicals (Slavin & Lloyd, 2012). This view is supported by the literature which associates low intake of fruits and vegetables with chronic diseases such as hypertension, cardiovascular diseases, hypercholesterolemia, osteoporosis, different types of cancers, and chronic obstructive pulmonary diseases among others (Pem & Jeewon, 2015).

Some commentators and researchers have reported on the impact of the pandemic on food choices (Ammann et al., 2022). On one hand, healthier food choices, i.e., a decrease in the consumption of sweet snacks and an increase in the consumption of vegetable consumption were reported for some individuals who were in a position of working remotely. On the other hand, unhealthy food choices and lifestyles, i.e., a reported increase in alcohol consumption and an increase in sweet snack consumption were also reported to be the consequence of remote working. Individuals who worked remotely were more likely to gain or lose weight than individuals working from the office.

All aspects of the population's daily life and routines were affected by the introduction of several policy measures to contain the pandemic. This resulted in an impact on individuals' everyday life with health‐related behavior (such as dietary habits and lifestyle factors) being the most affected (Hassen et al., 2021). The wider sociological aspects of food behavior have been recognized as key features of dietary patterns associated with better health outcomes (Georgousopoulou et al., 2017). However, several social restrictive measures (lockdowns) disrupted normal social functioning linked to a range of features of what could be normal food behaviors. Even though many households ate together much more than usual during the pandemic, households were not allowed to mix, and this limited the sharing of food with others. Additionally, the number of people who could attend celebrations such as weddings and birthdays among others was restricted and consequently, the opportunity to share food was also restricted. The restrictions in being able to share food with others should not be dismissed as a trivial minor effect of the restrictions. The impact of this which resulted from societal and legislative responses to the pandemic virtually eliminating the cultural norm of eating with others, which is recognized as a common theme across all cultures globally (Rozin, 1996). The sharing of food with others is a feature central to most faiths, especially feasts and celebrations, and the forced abstinence from the social sharing of food should be seen as perhaps an underlying factor compounded by the other disruptive impacts of the pandemic on food supply. The impact of social restrictions seen during the pandemic could be seen as being similar to the protective measures seen in war zones and during climate disasters and fires. Although generally these are for shorter periods in the case of climate disasters and fires. The importance of food‐related social interactions has been recognized and has been included in national food guidelines, including Brazil (Brazil, 2015) as well as being suggested in others, e.g., as part of the cultural features of the Mediterranean Diet (UNESCO, 2013).

The disruption to the supply chain and any accompanying hoarding or panic buying because of the pandemic resulted in limited access to fresh foods as well as nonperishable foods that would be part of a healthy diet such as canned pulses, fruits, and vegetables, which could have increased the reliance on unhealthier foods which can tend to have a longer shelf life (Robinson et al., 2021). With the hoarding of nonperishable foods, which can tend to be the less healthy options, the risk of dietary patterns becoming less healthy was, therefore, increased. Additionally, the limited availability of fresh foods was shown to result in changes in eating habits. Emerging evidence has shown mixed effects of the pandemic on eating habits with some authors suggesting an increase in snacking and unhealthy eating habits during confinement (Ammar et al., 2020). On the contrary, other authors conducted studies that reported reductions in the consumption of unhealthy foods such as snacks, cakes, cookies, and pastries (Di Renzo et al., 2020; Hassen et al., 2021), and higher vegetable consumption (Murphy et al., 2020). Food choice or changes as a result of the pandemic were suggested to be the result of several factors including (i) A reduction in the consumption of fresh food was found in most households except for those with children (Janssen et al., 2021), (ii) The level of education was also a major determinant and contributor to food choices and this impacted the quality of diet (Ammann et al., 2022). Positive changes in food choices were reported for individuals with higher levels of education, which was supposed to be related to increased opportunities to work from home (Jaeger et al., 2021). On the contrary, Poelman and co‐workers reported the opposite effect with higher individuals who were highly educated reporting unhealthier diets during lockdown than individuals with lower education levels (Poelman et al., 2021). (iii) Age was also documented to be a major factor influencing the dietary intake. A study by Rolland and colleagues showed no significant difference in dietary habits of older adults (Cicero et al., 2021). About 50% of older adults who were 50 years and older did not increase their intake of calorie‐rich or salt‐rich foods; and no significant changes in diet or alcohol consumption were reported (Rossinot et al., 2020). A decrease in alcohol consumption was reported in some studies (Callinan et al., 2021) whereas other studies (Cicero et al., 2021) showed an increase in alcohol consumption where, for instance, about 69% increase in alcohol consumption was reported during the pandemic in Italy which happened to be one of the countries to be hit the most with the pandemic (data collected as part of the ongoing Brisighella Heart Study longitudinal population study). The increase in the consumption of alcohol was linked to an increase in the level of stress and thus alcohol was seen to be a coping mechanism. Alcohol consumption has been identified as a significant risk factor for illness, disability, and mortality (Rehm, 2011).

Over 800 million people are reported to be facing chronic hunger and over 100 million people are facing acute severe food insecurity. Thus, these groups were the most affected by the disturbance in food access brought by the pandemic (Poudel et al., 2020). Levels of severe hunger have spiraled since the pandemic with the number of people facing famine‐like conditions being shown to increase considerably reaching over 500,000 people across, countries such as Ethiopia, Madagascar, South Sudan, and Yemen (Oxfam, 2021). It is important to note that most of the countries that are experiencing catastrophic levels of hunger have witnessed protracted periods of conflict, insecurity, and violence. The impact of conflicts and war on nutrition will be discussed at a later stage in this review.

### 2.2 Food options, choice, and behaviors in conflict‐affected zones

Food insecurity is a complex problem that may arise from many factors that include changes in demography, failure in institutions and governance, poverty, climate change and natural disasters, and conflict among others (Swesi et al., 2020). In recent years, conflict has been identified by the United Nations to be a major driver of food insecurity as it is the major contributor to the increase in global food insecurity with estimates showing that between 720 and 811 million people in the world faced hunger in 2020. Conflict alone has resulted in a figure of 795 million people facing hunger (Von Grebmer et al., 2016), and the number of people facing food crises or worse continues to increase each year.

Studies on South Sudan have shown people in conflict zones to rely on less preferred and less expensive foods. An increase in the consumption of green leafy vegetables collected in the open field as a wild crop was also reported to be a coping strategy in South Sudan for people affected by conflict (Sassi, 2021). Moreover, this was reported to be the only source of food for poor and vulnerable households during the hunger season. Green leafy vegetables are consumed for satiety reasons. However, a diet that is mainly composed of one food group is far from being nutritious and sufficient to provide the recommended dietary values for some important nutrients and minerals such as vitamin A. Insufficient consumption of such nutrients as vitamin A is one of the causes of death and disease in war‐torn countries country, especially among children (Katona & Katona‐Apte, 2008). Additionally, the consumption of a diet composed of one food group can result in calorie deficit and this could increase the risk of moderate and acute malnutrition.

The other factor that has affected the availability of food is the impact of Russia's invasion of Ukraine. Ukraine and Russia are major food exporters of the leading cereal crops (such as wheat, maize (corn), and barley) as they each provide about 6% of global market shares in food calories (Weil & Zachmann, 2022). As the war escalates, there have been several food storage facilities that have been destroyed, along with significant disruption to logistics and distribution as a result the export of foods has been impacted with the aftermath being felt in several countries, particularly in Africa (e.g., in Ethiopia, Nigeria, and South Africa) (Eziakonwa, 2022) as well as around the globe.

As the devastation in Ukraine continues to unfold, there have been many warnings about the global food crisis precipitated by the war with an increased focus on the risks of famine and severe food insecurity (Osendarp et al., 2022). Women and children are the most affected by the food shortages and high food prices resulting from the war against Ukraine (Osendarp et al., 2022). They are especially vulnerable to malnutrition given that children's nutritional needs are high relative to their body size, and women's are high especially when pregnant or lactating. Moreover, it has been suggested that existing power imbalances and gender inequalities which tend to be exacerbated during crises mean that women have less agency to direct resources toward feeding themselves and their children (Marphatia et al., 2016). Notwithstanding, these groups have already been disproportionally affected by the combined effects of other conflicts, climate change, and the COVID‐19 pandemic. Even though the impact of malnutrition might be less immediately visible than that of hunger if left untreated, malnutrition can increase the risk of illness and death in the short term, and in due course have multigenerational and irreversible health effects (Osendarp et al., 2022).

The other impact of conflicts and war is starvation. The starvation of civilians is an all too frequent feature of armed conflict. Even though starvation may occur as an unintended consequence of military activities, there have been some reports that have shown this to be sometimes intentionally used by conflicting parties as a method of warfare (D'Alessandra & Gillett, 2019). Starvation has been shown to have an impact on the human body with the diversity and richness of the gut microbiota being shown to be altered by prolonged starvation (Mack et al., 2018).

### 2.3 The impact of climate change on global food availability, food supply, food choice, and food security

There is an expectation that climate change will increase the intensity, frequency, as well as spatial extent of extreme climate events, and as such is a key concern for food production (Dasgupta & Robinson, 2022). The variability in climate accounts for approximately 30% of the variability in global agricultural yields, which in turn increases the uncertainty of food production and prices across various geographical scales, consequently threatening food security (Verschuur et al., 2021). The climate‐induced risks to food insecurity are often driven by exposure to extreme climates such as extreme droughts, and the susceptibility and reaction of the food‐supply system to food production shocks (Ziervogel & Ericksen, 2010). Reduced food production and a concomitant spike in food prices are often felt unduly by poor consumers, who in most cases do spend a larger share of their household budget on staple foods (Brown & Funk, 2008), and this can potentially lead to them forfeiting the consumption of these foods or being pushed into poverty (Headey, 2012).

The production of food is affected by climate change, and, in turn, food production is responsible for a quarter of anthropogenic greenhouse gas emissions globally (Parker et al., 2018). As the world population increases, there is an increase in the demand for food production that must meet nutrition and health needs while simultaneously assisting in achieving the sustainable development goals. Climate change has a significant effect on the food supply, and this can affect both access and availability. As such, there is a need for changes to be made to food production, with the nutrient content being monitored, and more equitable distribution is needed to meet the dietary guidelines.

The magnitude of the impact of climate change on the food supply has increased over time. Some authors that posit that climate change “has been responsible for reversing some of the improvements in food security that would otherwise have been realised, with the highest impact in Africa” (Dasgupta & Robinson, 2022). Changes in precipitation have been reported to be responsible for the reduction in consumable food calories. The link between climate change and food production (Ray et al., 2015) has also been identified. For instance, a link between long‐term droughts, high springs, summer precipitation and warm winters, and the outbreak of locusts that can be devastating for crop production has been found (Dasgupta & Robinson, 2022). However, it is important to note that there is still a gap in the literature with respect to the identification of a plausible causal relationship between climatic stressors and food security indicators.

Food shortages and famines have reoccurred throughout human history which is mainly due to a variety of interrelated causes that include environmental crises and climate changes due to the global warming effect of greenhouse emissions, natural disasters, socioeconomic and political inequalities, and violent conflicts (Baro & Deubel, 2006). Several countries such as China and the Netherlands have experienced famine in the past. An unprecedented famine was experienced in China in 1959–1961. Insufficient food supply resulted in the lack of the calories that are needed for survival, and famine eventually occurred (Johnson, 1998; Kung & Lin, 2003). Due to the enemy's invasion, the Western Netherlands was exposed to severe famine between September 1944 and May 1945. As a result, the population was reduced to starvation rations (Hart, 1993). The percentage of the world's population facing acute and chronic hunger has been shown to decrease on every continent except Africa (Brown, 2001). Sub‐Saharan Africa remains to be one of the regions of the world where chronic food insecurity remains endemic. Additionally, threats of famine remain endemic for most of Africa's population and the number of malnourished people is steadily increasing (Otekunrin et al., 2020). The main consequences of famine are a concentrated decline of food consumption, which results in chronic underweight for individuals, including stunted growth and development in infants and children, as well as a sharp increase in excess mortality, in addition to a major social disruption, and long‐term depletion of resources.

The relationship between access and availability of food is codependent, which means that if food is not available, neither is it accessible. While plenty of food can be available, many different factors might hinder access to it. Several global events such as conflict, pandemics, and climate which have all been discussed in this paper have been shown to have the potential of disrupting the availability and accessibility of food. Thus, to mitigate against their impact on the global food supply, there is a need for the food industry to think of ways of diversifying food production and making the food supply chain more resilient and this will reduce the adverse effects of such disruptions on food security.

## 3 THE IMPACT OF THE THREE LETHAL C'S ON THE COST OF LIVING AND HOW THIS CRISIS IMPACTS CONSUMER SHOPPING AND DIETARY HABITS AND BEHAVIOR

The cost‐of‐living crisis can be the result of three factors – COVID, conflict, and climate change. An economic crisis can have a significant negative shock on consumers and affect the macro‐ and microlevel economy (Basen, 2014). Some of the major impacts of the crisis include a rise in the cost of living, no change in wages, lower working hours, and unemployment. Consumers' response to an economic recession in food purchasing and consumer behavior has been researched (Basen, 2014; Muresan et al., 2022). There is a general tendency for food prices to increase due to inflation and food companies changing their market strategies to increase the price per quantity of foods and package content (Jenkins et al., 2021). Unlike other global events, economic crises due to limited resources by their very nature reduce purchasing function and do not tend to be associated with hoarding or panic buying of nonperishable food. However, the preparation of healthy meals and food shopping are all impacted by household income. The interconnection between the consumption of food products and income is strongly affected by the level of per capita income (Milford et al., 2019). This impacts not just the ability of consumers to purchase foods, but it can also limit the ability to prepare and cook food due to potentially increased fuel costs along with energy costs associated with cleaning used food preparation equipment. Consumers with a low income generally allocate the majority of their disposable income to food consumption and are most likely to spend a large amount of additional income on food, and this does result in a significant impact on food consumption (Fukase & Martin, 2020; Rask & Rask, 2004). On the contrary, the ratio of income allocated to food consumption falls sharply with income in higher income consumers. Consequently, the effects of income growth on food demand are weakened. Households with a higher income can consider factors such as convenience and quality when making food choices, resulting in these decisions being easier and requiring less psychological and sociological ‘work’. Packaging, advanced preparation, and away‐from‐home consumption are also major considerations for high‐income consumers (Basen, 2014). With a squeeze on income, consumers typically move toward various kinds of products, such as low‐quality items and processed foods. Additionally, food products must compete to share their balance of the food budget, this can lead to a strong need to substitute or replace items (Wijesinghe & Kaushalya, 2022). As such, there tends to be an increase in consumption of some products while others diminish in consumption. Some consumers will tend to buy more products which are perceived to be cheaper and necessary in an economic recession with the consumption of products such as meat (Sane Schepisi et al., 2021) and fish (Alves & Perelman, 2019), which are seen to decline due to their higher prices. Meat and fish offer a higher protein content, which is also of high biological value meaning they have a higher anabolic effect, bioavailability, and digestibility compared to proteins that are obtained from plant sources. This is mainly attributed to their lower essential amino acid content (especially leucine). Furthermore, plant storage proteins have been reported to be deficient in other essential amino acids, including sulfur‐containing lysine and methionine (Berrazaga et al., 2019). Additionally, the nutritive value of plant storage proteins is reported to be inferior to that of animal storage proteins (Munialo & Andrei, 2023; Munialo & Vriesekoop, 2023; Munialo et al., 2022), perhaps linked to the matrix of the food product making digestion and bioavailability more challenging.

A shift toward processed foods that can be deemed cheaper can also be the result of an increase in the price of foodstuff and this can have an impact on health. The decision to shift toward processed foods is often balanced alongside additional costs of preparation and cleaning up along with the risk of food not being consumed (especially in families) to minimize waste. This can have the result of encouraging an increase in those living on a lower income choosing less fresh food (minimally processed foods) and more ultra‐processed foods (UPF). This potentially is associated with increased future health and societal costs, given that an increase in evidence suggests that the high consumption of UPF is associated with an increase in noncommunicable diseases, overweight, and obesity. Furthermore, several authors have shown a high UPF consumption results in a significant increase in the risk of cardiovascular diseases, depressive symptoms, type 2 diabetes mellitus, and cancer (Adjibade et al., 2019; Fiolet et al., 2018; Srour et al., 2019, 2020).

Besides being the motivation to reduce waste and be certain that food is eaten, an economic downturn can also have a significant impact on negative emotions, such as increased feelings of depression and stress due to unemployment or loss of employment, the fear of being made redundant, and boredom due to joblessness. This can have a concomitant effect of adopting unhealthy diets and less healthy food choices (Ben Hassen et al., 2021). Psychological reactions to unemployment stress (including perceived risk of unemployment) or stress due to increased living costs where households are forced to make decisions between food and bills, often termed as the choice between ‘eating and heating’, with a struggling economy can result in disrupted diets, and this can lead to unhealthy eating patterns and frequent snacking. Negative emotions, such as stress and anxiety, can lead to overeating, “emotional eating,” especially the so‐called “comfort foods” (typically foods high in fat and sugar, such as chocolate and ice cream), and frequent snacking. This could compound the drive to increase the consumption of UPF. Emotional eating has been associated with weight concerns such as overweight and obesity (Dohle et al., 2014; Frayn et al., 2018). Individuals with overweight have been found to show less effective coping abilities when it comes to responding to negative emotions, and this leads to an increase in the frequency of emotional eating (Godet et al., 2022; Ozier et al., 2008). Difficulties with weight loss have also been associated with emotional eating (Butryn et al., 2009; Delahanty et al., 2013; Frayn & Knäuper, 2018).

## 4 SOME STRATEGIES FOR MAINTAINING A HEALTHY DIET DESPITE CHANGES IN THE GLOBAL ECONOMY, ENVIRONMENT, AND/OR POLITICAL SYSTEMS

In most cases, when it comes to adopting healthy eating, there needs to be a shift and/or change in behaviors. Many theories have been put forward that show how behavior changes affect health. Health promotion requires people to initiate and maintain healthy behavior changes, which is an integral part of the integrated theory of health behavior change (Ryan, 2009). Thus, for individuals especially in those vulnerable households to be able to maintain healthy eating, there is a need for a shift in mindset and behavior change is needed which should provide resilience and preparedness to deal with unprecedented moments of a pandemic, war, and recession among others. Some of this will include changes in planning, shopping, cooking, and serving of foods as well as a rethink on the perception of some foods, which does affect food choices.

When faced with the choice between food and bills, in some cases, food becomes neglected even though it is one of the major human needs. In most cases, some people may think that healthy foods are expensive and as such would resort to eating processed or “junk” food. However, having an idea of how to combine some of the existing foods to enhance their nutritional value is vital. With the soaring prices of meat and fish products, consumers can resort to decreasing the frequency of consumption of these products within the household. Households should not shy away from the consumption of canned foods given that canned fruits and vegetables have been reported to be nutritious options available year‐round at a competitive cost to fresh and frozen ones (Miller & Knudson, 2014). In high‐income countries, households who need assistance can use food banks including food pantries and social supermarkets that provide free groceries.

Food waste is a major issue when it comes to the availability of nutritious meals to feed the population. There exists a paradox when one thinks of the fact between 7750 and 15,345 people die each day from hunger and malnutrition (Oxfam, 2021), whereas nearly one‐third of food which equals to a total of 1.3. billion tons of food per year is wasted globally (Gustavsson et al., 2011). As such, households need to come up with ways in which they can minimize food waste and this could include the revaluing of leftovers as a conduit for disposing of surplus food. The use of leftovers should give the food a ‘second chance’ rather than this food being thrown into the bin (Cappellini & Parsons, 2012). Additionally, consumers should opt to buy and use the food that they need rather than allowing things like promotions and offers to result in the purchasing of foods that were not intended which ended up not being consumed and instead are wasted.

Another strategy is adopting some changes in the composition of the households' shopping basket. For instance, households can decide to switch from a preferred brand to a cheaper generic product which still has nutritional quality (Griffith et al., 2016) but will be cheaper than branded products. Individuals can also choose to become more open to the consumption of emerging (alternative) sources of food proteins such as insect proteins, mycoproteins, and the emerging plethora of plant proteins (such as pea, lupins, sunflower, mung bean, and potato among others) (Lo et al., 2021; Munialo et al., 2014, 2018, 2022). Insect proteins have been reported to be nutritious and sustainable protein sources and this can be used as part of the multifaceted strategies to bolster the fragile food supply to feed individuals during times of scarcity (Kouřimská & Adámková, 2016). It is generally suggested that edible insects can provide ecological and economic advantages in addition to their nutritional benefits (Patel et al., 2019) although there is a low consumer acceptance of edible insects compared to conventional animal or plant food sources (Kim et al., 2019).

Malnutrition is often viewed predominantly as a problem of hunger resulting from the lack of food or an imbalance in food distribution at the community and household levels and this is often attributed to food insecurity. In areas where the cost of living becomes a hindrance to the consumption of a healthy diet, there are several strategies that can be used to improve access to nutritious food, especially in individuals from low socioeconomic status. An example can include educating vulnerable households to engage in the growing of their fruits and vegetables such as salad leaves, carrots, and cucumbers among others. Such crops do not require large pieces of land to produce. Additionally, growing some of the vegetables requires minimal skills to grow. This can be a good and great initiative, e.g., for households to also engage children in the planting and caring for the crops but also the produce can contribute toward healthy family meals.

It is worth noting that there is a diversity of causes of food insecurity and so are some of the proposals that exist in trying to combat the issue. For instance, several proposals have been considered in high‐income countries such as the UK. These proposals included help with fuel costs, increased benefits, and the extension of the provision of free school meals and holiday food. However, in low‐income countries, many people resort to coping strategies such as minimizing the number of meals, rationing the amount of food consumed, and out‐migration of household members during chronic food shortages. Given the disparity that exists in food insecurity among the different nations, the global community must see access to food as a key human right. Although no hunger is specified as the second sustainable development goal (SDG) (SDG, 2019), the impacts of the social stressors and disasters described in this paper overarch most if not all of the SDGs. It is important that governments respond to the recognition that food supply is more than malnutrition and having enough calories to survive and that at times of conflict and disaster, it is essential that more than adequate calories need to be the goal. If this can be achieved, then the harm and trauma of such events would at least be mitigated with respect to their impact on long‐term health.

## 5 CONCLUSION

Several factors such as war, pandemics, and economic crises have the potential to impact negatively on global food security due to disruption in the global food supply chain. Consequently, the price of food commodities will increase and this makes it difficult, especially for households with a low income, to access food products. As such, some households may resort to unhealthy food choices as a means of survival. However, with proper education, as well as the implementation of several strategies (at a household level) such as buying supermarkets' own brands, buying canned foods, as well as growing vegetables, and food banks which include social supermarkets and food pantries can be used to ensure that households, and in particular the vulnerable ones can have nutritious meals despite the constraints of which they will have little or no control. At a global scale, there needs to be several coordinated policies that ensure access to healthy diets and foods such as where governments invest in programs that allow for the continual production and distribution of foods even in times of restrictions or conflicts. The food industry also needs to come up with processes and food production practices that are resilient to the ever‐emerging issues that have the potential of disrupting the food supply chain. These measures need to align with the UN‐SDGs.

## AUTHOR CONTRIBUTIONS

**Claire D. Munialo:** Conceptualization (lead); data curation (lead); project administration (lead); writing – original draft (lead); writing – review and editing (lead). **Duane D. Mellor:** Conceptualization (supporting); writing – original draft (supporting); writing – review and editing (equal).

## CONFLICT OF INTEREST STATEMENT

The authors declare that they have no conflict of interest.

## ETHICS STATEMENT

Ethics approval was not required for this research.

## Data Availability

Research data are not shared.
